# “Model T” Cells: A Time-Tested Vehicle for Gene Therapy

**DOI:** 10.3389/fimmu.2013.00304

**Published:** 2013-09-27

**Authors:** Sid P. Kerkar

**Affiliations:** ^1^Laboratory of Pathology, Center for Cancer Research, National Cancer Institute, National Institutes of Health, Bethesda, MD, USA

**Keywords:** gene therapy, cancer, immunotherapy, T cells, inflammation, chimeric-antigen receptors, cytokines, severe combined immunodeficiency

## Abstract

T lymphocytes first carried foreign genes safely into humans over two decades ago. Since these pioneering studies, scientific techniques to better understand the genomic landscape of cells has directly led to a more sophisticated appreciation of the diversity, functional complexity, and therapeutic potential of T cells. Through the use of mouse models, we now know the function of the many genes that are critical for T cells to recognize foreign, mutated, or self-antigens and the factors responsible for the lineage diversification of T cells that lead to inhibitory or stimulatory immune responses. This knowledge combined with well-established modalities to introduce genes into T cells allows for the design of effector and memory CD8 and CD4 T lymphocytes specific for viral, fungal, bacterial, parasitic, and tumor-antigens and to design regulatory lymphocytes specific for the self-antigens responsible for autoimmune and inflammatory diseases. Here, I review strategies for designing the ideal T cell by introducing genes controlling (1) the secretion of cytokines/chemokines and their receptors, (2) T-cell receptor specificity, (3) chimeric-antigen receptors that enable for the recognition of surface antigens in an MHC-independent fashion, (4) co-stimulatory/inhibitory surface molecules, and (5) disease defining single-gene factors.

## Introduction

The Deoxyribonucleic acid (DNA) molecule, perhaps one of biology’s greatest discoveries, helped unlock the secrets to the flow of genetic information that we now know forms the basis for the complexity of all life on earth. In the 1960s, the scientific community demonstrated for the first time that exogenous DNA could be taken up and ectopically expressed in mammalian cell lines (1). Shortly after, a growing understanding of viral reverse transcription processes and advances in recombinant DNA technologies paved the way for engineering viruses to carry therapeutic genes into cells (2).

Fast-forward 40 years and there now exists numerous viral and non-viral modalities to introduce therapeutic genes into cells. The most common viral vectors include retroviruses, adenovirus, and herpes simplex viral backbones with non-viral modalities centered on physical (DNA transfection/electroporation) or chemical (synthetic oligonucleotides, lipoplexes, nanoparticles) methods of delivery and transposon systems (3–6). Of these various modalities, gene therapy using retroviral based vectors is perhaps the most established methodology both in experimental models and in human clinical trials due to the ability to stably integrate genes into dividing cells (7–9).

In addition to the technologic advancements in gene therapy, a growing understanding of the genetic causes of human disease and the downstream function and network-like interactions between specific genes are enabling scientists to devise strategies to treat ailments once thought incurable (10, 11). While the *in vivo* delivery of genes targeting specific cell types remains a grand hope for the future, current methodologies readily enable for the stable introduction of foreign genes into cells *ex vivo*, allowing for the transfer of these cells back into patients (6).

T lymphocytes represent the ideal vehicle for carrying therapeutic genes into humans. T cells are easily obtained through peripheral blood draws or apheresis procedures and can be induced to divide robustly *ex vivo*, a characteristic that allows them to be highly permissible to retroviral introduction of ectopic genes (12). The first clinical trial to safely infuse a foreign gene into humans consisted of transducing tumor-infiltrating lymphocytes with a neomycin resistant gene that enabled for the detection of the transgene within a tumor biopsy several days following transfer (13). Today, the adoptive transfer of tumor-infiltrating lymphocytes combined with total-body irradiation, lymphodepleting chemotherapy, and high-dose IL-2 achieve response rates as high as 70% in patients with metastatic melanoma (14). The rapid development of gene therapy in this field promises to vastly improve current cellular therapies and opens the door to treat cancers of various histologies and wider arrays of human disease. Here, I discuss potential therapeutic genes that may improve current gene therapies, although rigorous pre-clinical testing and careful phase 1 clinical trials will be required for many of the suggestions in this review.

## Cytokines, Chemokines, and Their Receptors

The theory of immune surveillance in cancer is controversial but there exists reproducible scientific data pointing to the importance of interferon-gamma as a critical mediator for the elimination of malignantly transformed cells (15). Furthermore, there is a clear increase in the incidence of cancer in patients with HIV, Immunodeficiency syndromes triggered by mutations in genes such as GATA-2 (MonoMAC) and post-transplant patients receiving immune-suppressive drugs (16, 17).

Additional support for the importance of an immune response for cancer elimination can be garnered from clinical data with robust long-term follow up showing the ability of systemic IL-2, anti-CTLA-4 antibodies, and anti-PD-1/anti-PD-L1 antibodies, and the adoptive transfer of T lymphocytes to induce tumor regression in patients with metastatic melanoma and metastatic renal cell carcinoma (18, 19). Three major factors are important for an effective immune response against cancer: (1) overcoming suppressive factors induced by mutated cancer cells within the tumor microenvironment, (2) the quality of the T cells transferred, and (3) polymorphic factors of an individual’s host immune response. Some of these factors can be readily modified by over-expressing cytokines, chemokines, and their receptors in transferred T cells, enabling lymphocytes to secrete supra-physiologic amounts of therapeutic immune-stimulatory molecules.

### The IL-12/IFN-γ/TH-1 axis

IL-12 is a hallmark inflammatory cytokine and is critical for driving an effective immunologic response against cancer and foreign pathogens (20). It is mainly produced by inflammatory cells such as dendritic cells, macrophages, and neutrophils and directly augments the functionality of multiple end effectors such as CD4^+^ T cells, CD8^+^ T cells, natural killer (NK) cells, and NKT cells (20). The anti-tumor effects of IL-12 are well documented (21). IL-12 enhances the ability of CD8^+^ T cells to cause the regression of large established tumors by potently stimulating the production of high-levels of IFN-γ, resulting in an increase in the cross-presentation of tumor-antigens and the reversal of suppressive functions of myeloid-derived suppressor cells, alternatively activated macrophages, and dendritic cells (22). These changes subsequently lead to the collapse of the tumor stroma and the regression of large established masses (22, 23).

**Figure 1:**
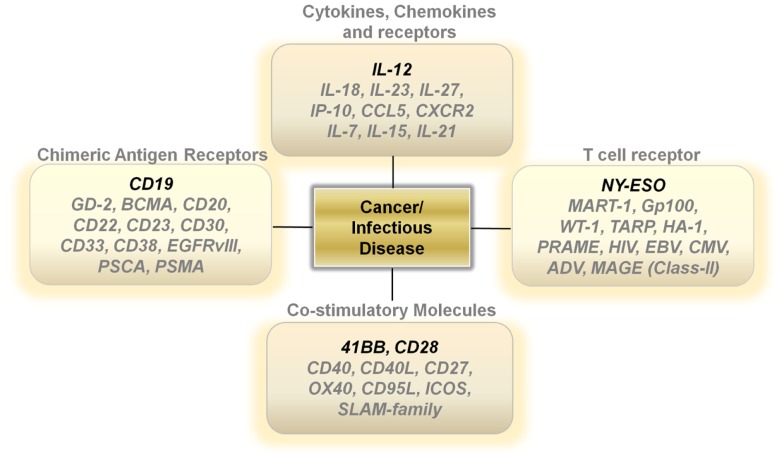
**Potential therapeutic gene therapies in T cells for Cancer and Infectious Diseases**.

Unlike activated lymph nodes stimulated by pathogen-activated molecular patterns, sterile conditions within tumors lead to low levels of IL-12 secretion by innate immune cells. This lack of a danger signal within the tumor microenvironment results in a skewing away from a Th-1 type effector immune response. One attractive approach is to increase the levels of IL-12 directly at the point of T-cell/Tumor cell and T-cell/Antigen-presenting contact within tumors (24) (Figure 1). Several studies show that over-expressing a single-chain, functionally active IL-12 gene in tumor-antigen-specific lymphocytes significantly increases the levels of IL-12 to supra-physiological levels within tumors, leading to the regression of large established masses (25–28). This modification enables for therapeutic anti-tumor immunity with smaller numbers of T cells and does not require the use of systemic gamma-chain cytokines to support the transfer of cells *in vivo*. Currently, clinical trials are determining if the benefits of IL-12 gene therapy outweigh the many risks associated with a systemic increase in IL-12 and IFN-γ.

### Additional cytokines, chemokines, and chemokine receptors

The importance of gamma-chain cytokines in the proliferation and maintenance of memory T cells remains a critical and extremely important avenue of research for many investigators (29–32). However, clinical trials using TIL transduced with the IL-2 gene did not show a clinical benefit (33). Over-expressing the other gamma-chain cytokines such as IL-7, IL-15, or IL-21 in T cells may lead to better results. However, the constitutive expression of genes that drive T-cell proliferation carries the risk of causing an uncontrollable expansion of transferred T cells due to the stable integration of retrovirally transduced genes being expressed in every daughter cell (Figure 1). Designing viral vectors using a NFAT promoter for inducible gene expression upon T-cell receptor (TCR) ligation may provide an important degree of safety (26). Another strategy is to use adenoviral vectors or systems that only transiently express the genes that control T-cell memory formation.

Other potentially attractive cytokines include those within the IL-12 family, such as IL-23, and IL-27 (Figure 1). These cytokines may invoke beneficial downstream mechanisms for anti-tumor immunity without the heavy reliance on the induction of IFN-γ secretion. Furthermore, genetic polymorphisms within the human population may make certain individuals more likely to mount an anti-tumor response to one of the alternate members of the IL-12 family rather than IL-12 itself.

Another strategy that may turn out to be fruitful is the over-expression of chemokines and chemokine receptors in T cells (Figure 1). Melanomas can secrete chemokines such as CXCL1 and CXCL8 to aid in the recruitment of monocytes into local microenvironments and studies show that expressing the chemokine receptor CXCR2 on transferred T cells aids in the ability of T cells to infiltrate tumors and cause regression (34). This approach can be easily tailored to other tumor histologies depending on the chemokine secretion profile of the cancer. Additionally, over-expressing chemokines in T cells may also provide some benefit. Upon recognizing cognate antigens, T cells arrest their migration and accumulate at sites with productive antigen presentation. The over-expression of chemokines such as IP-10 or CCL5 in transferred T cells may enable antigen-specific T cells to secrete products that attract activated T cells to the local microenvironment they inhabit. This in turn may provide a positive feedback loop that enables for an increase in infiltrating antigen-specific T cells and an improved therapeutic outcome. Thus, the possibility to genetically alter the cytokine or chemokine profile of adoptively transferred T cells may prove to enhance and simplify current treatments requiring lymphodepletion and high-dose IL-2.

## Chimeric-Antigen Receptors

The ability to generate a single fusion molecule that can bind surface antigens and trigger T-cell function holds great promise for the future of cell therapy. Chimeric-antigen receptors (CAR) are the latest form of gene therapy, where a single vector is constructed with a binding moiety recognizing a surface antigen [usually designed from a single-chain variable fragment (scFv) derived from a tumor-antigen-specific monoclonal antibody] (35–37). The beauty of CAR generated T cells is the ability to generate lymphocyte specificity in an MHC-independent fashion due to the ability to design receptors that recognize surface antigens. This is accomplished by cloning the sequences from the variable region of antibodies (many of which already exist) and adding T-cell signaling and co-stimulatory domains to the vector construct.

Early phase trials for CARs recognizing the antigen CD19, expressed on many B cell lymphomas and leukemias are showing promising results in adult and pediatric patients at multiple institutions (38–41). One of the major advantages of using CARs as the main platform for gene therapy is the ability to rapidly and clearly define the expression of the target protein. Often, the antibodies whose variable region is cloned into the CAR vector can also be used diagnostically to look for the expression of the desired target.

Other antigen targets that may be worthwhile exploring for CAR development includes GD-2 for neuroblastomas (42), CD20 (43), and CD22 for B cell lymphomas (44), BCMA (B cell maturation antigen) (45), and CD38 for multiple myeloma (46), CD23 for chronic lymphocytic leukemia/small lymphocytic lymphoma (CLL/SLL) (47), CD30 for Hodgkin’s lymphoma and anaplastic large cell lymphomas (48), CD33 for acute myeloid leukemias (49), EGFRvIII for glioblastomas (50) and PSCA (51) and PSMA (52) for prostate adenocarcinomas (Figure 1).

## T-Cell Receptors

Although CAR-directed gene therapy remains a promising modality for the future, many cancers, especially carcinomas and sarcomas, do not possess known surface expression of unique non-shared antigens. Targeting surface proteins that may be expressed on normal tissue with CARs may cause serious end organ damage and toxicity. Gene therapy using high avidity TCR enables for the design of lymphocytes targeting epitopes from differentially expressed or mutated intra-nuclear and/or intra-cytoplasmic proteins such as transcription factors (22, 53). Emerging data now shows that tumor-infiltrating lymphocytes possess the ability to recognize mutated melanoma antigens (54, 55). This exciting finding opens up a large window of opportunity to develop effective TCR gene therapies. It is possible that in the future we may perform whole exome sequencing for every tumor for diagnostic purposes, enabling us to design TCR recognizing the most frequently mutated epitopes for different tumor histologies.

A great example of the success of TCR gene therapy was recently described with a clinical trial utilizing the NY-ESO TCR (56). This study led to significant tumor regression in four out of six patients with synovial sarcoma and five out of 11 patients with metastatic melanoma. Overall, the cancer-testis antigens represent an ideal set of target antigens due to their relatively low to negligible expression on normal tissue, except in the testis, where cells express low levels of MHC Class I. Identifying antigens with limited normal tissue distribution will be critical to extending TCR gene therapy to different types of cancer. Developing TCRs for breast, prostate, and thyroid cancer also seems reasonable since targeting of normal tissue in these organs might not be accompanied with serious life-threatening adverse events (Figure 1).

## Co-Stimulatory Molecules

Generating both a specific and a productive T-cell response requires not only appropriate signaling through the TCR but an additional secondary co-stimulatory signal. The most well studied co-stimulatory molecule is CD28, a disulfide linked homodimer that is constitutively expressed on naive T cells (57). CD28 engagement with CD80 and CD86 on antigen-presenting cells enables T cells to differentiate and become functionally activated (57). However, after initial antigen encounter and under altered cytokine conditions, T cells can lose or decrease their expression of CD28, leading to replicative senescence and functional anergy. The lack of CD28 signaling can also result in an impaired memory response and activation induced cell death (AICD) (58). One strategy to circumvent these physiological restraints is to constitutively over-express CD28 in T cells. Currently, second and third generation CAR constructs use the intracellular domain of CD28 to improve the persistence, function, and activity of CAR transduced T cells (59). Other important co-stimulatory molecules include 41BB, CD27, OX40, CD40, CD27, ICOS, Fas ligand, and the Slam family of proteins (60, 61). These molecules all have been implicated in tipping the balance in favor of generating a functional T-cell response and helping avoid AICD during antigen re-stimulation. The intracellular domains of 41BB, OX40, and CD27 are currently being incorporated into various CAR constructs that are being developed. Thus, it is possible that the constitutive over-expression of these various co-stimulatory molecules may aid in designing long lived, functionally active T cells that are resistant to cellular senescence (Figure 1).

## Severe Combined Immunodeficiency Syndromes

The first successful therapeutic gene therapy in humans in the early 1990s involved treating two children with severe combined immunodeficiency syndrome (SCID) caused by a genetic defect in the enzyme adenosine deaminase (SCID-ADA) (62). This syndrome resulted in defective T and B cells, leading to debilitating recurrent opportunistic infections. A normal/wild type ADA gene, enabling for the production of a functional enzyme, was introduced into T cells and infused back into the patient. The results were striking, and for the first time in these patients, there was evidence for IgM antibody production and the detection of tetanus antibody in the serum following immunization (62). In one patient, approximately 20% of the circulating lymphocytes still expressed the retrovirally inserted gene 10 years following transfer (63).

Although these initial results led to heightened optimism, attempts to develop gene therapies for SCID-X1, a disease characterized by a defective common gamma-c cytokine receptor subunit, by retroviral transfer of the corrected gene into CD34+ hematopoietic stem cells, led to the development of leukemias in some patients (64, 65). These setbacks sent shock waves through the scientific and medical communities. We now know that retroviral vectors can result in insertional mutagenesis, although this phenomenon still remains poorly understood (66). Five out of 20 patients treated in trials carried out in London and Paris developed leukemias secondary to the expansion of clones containing vector integration near proto-oncogenes such as *CCND* and *LMO2* (65).

Despite the clear dangers of gamma-retroviral gene transfer into hematopoietic stem cells, transferring genes into T cells *ex vivo* appears to be much more resistant to oncogenic transformation. There now exists robust long-term follow up for over a 100 patients treated on various gene therapy trials utilizing *ex vivo* retroviral insertion of genes into T cells with no evidence of malignant transformation (9). The mechanisms for the differences in oncogenesis between transducing hematopoietic stem cells versus T cells is not well understood. Perhaps introducing genes into more differentiated cells that contain a vastly different genetic and epigenetic landscape from stem cells leads to retroviral integration away from oncogenes.

Currently, gene therapists are continuing to try to improve safety through vector design. One strategy gaining support includes creating self inactivating gamma (SIN) retroviral vectors and lentiviral vectors by deleting the U3 region in the 3′ LTR (67). This modification generates a pro-virus with defective transcriptional activity at both the 5′ and 3′ LTR end regions, preventing the possibility of transcriptionally activating cellular oncogenes near the site of viral integration. Importantly, an internal promoter will need to be designed to drive the expression of the desired transgene within the SIN vector construct. Additionally safety measures include the genetic modification of shorter lived cell populations or the use of suicide genes (68).

**Figure 2:**
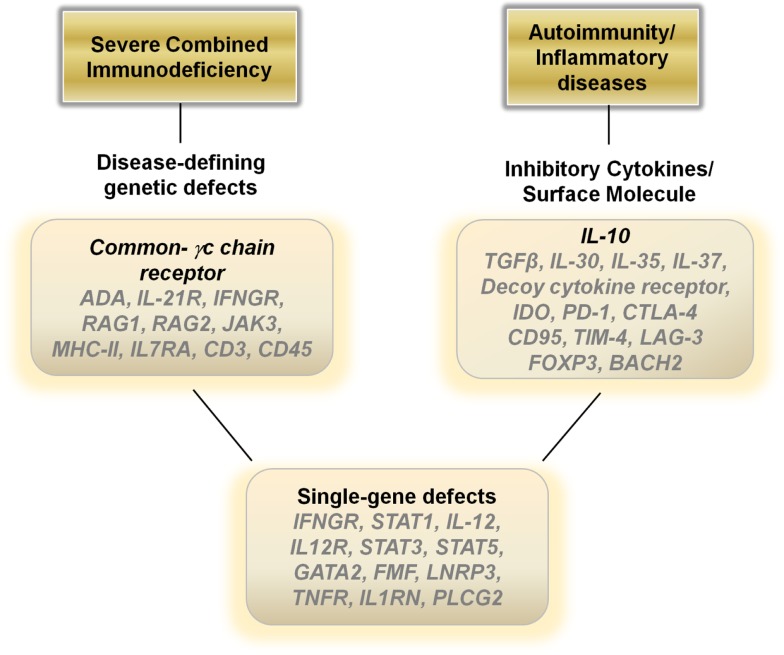
**Potential therapeutic genes to over-express in hematopoietic stem cells, monocytes, dendritic cells or T cells for autoimmune, inflammatory, and single-gene disorders**.

The current progress in improving the safety of gene therapies is helping the field move forward. In regard to SCID, although mutations in the common gamma-c chain receptor is the most common cause of the disease, a broad range of single-gene mutations can result in a similar disease pattern of recurrent opportunistic infections (Figure 2). Theoretically, for all the various defects that may occur, introducing a correctly functioning gene *ex vivo* into T cells or hematopoietic stem cells/monocyte/dendritic cell populations may re-capitulate the early excitement seen in the SCID-ADA trials and build on the recent successes of gene therapy for cancer.

## Autoimmune and Inflammatory Diseases

Although genetic modifications to stimulate the immune system is beneficial for battling infectious organisms and cancer, there also exists a set of devastating diseases that are caused by an over-zealous and unchecked immune response. The targeting of self-antigens under normal physiologic conditions can cause a range of serious ailments including type 1 diabetes, multiple sclerosis, rheumatoid arthritis, systemic lupus erythematosus, and autoimmune encephalomyelitis. Recently, a relatively new set of autoimmune diseases categorized as autoinflammatory diseases are beginning to be characterized such as familial Mediterranean fever (FMF), neonatal onset multisystem inflammatory disease (NOMID), tumor necrosis factor (TNF) receptor-associated periodic syndrome (TRAPS), deficiency of the Interleukin-1 receptor antagonist (DIRA) and Behcet’s disease (69, 70). Additional inflammatory diseases that cause morbidity and mortality in a large number of patients include inflammatory bowel disease (Crohn’s disease and ulcerative colitis), chronic granulomatous disease (CGD), and the various forms of vasculitis (71, 72).

In general, dampening the immune response is the ideal treatment for autoimmune and inflammatory diseases and current therapies revolve around the use of steroids, cytokine antagonists, or directly down regulating the immune system utilizing various modalities. Gene therapies may provide a viable biological alterative to directly blunt an over-active immune response. Over-expressing anti-inflammatory cytokines such as IL-10, TGF-β, IL-30, IL-35, or IL-37 in T cells or monocyte/dendritic cell populations *ex vivo* with a re-infusion of the modified cells may aid in decreasing inflammatory driven symptoms (Figure 2). Another alternative may be to construct a decoy cytokine receptor that contains the correct receptor sequence to enable for binding of pro-inflammatory cytokines such as IL-12 combined with a non-functioning cytoplasmic signal transducing sequence. Over-expressing these “dominant-negative” receptors would enable re-infused immune cells to function as sinks for the inflammatory cytokines responsible for the pathophysiology of the disease. Other genes that may aid in dampening the immune response include over-expressing indoleamine 2,3-dioxygenase in monocytes/dendritic cells or *CTLA-4*, *PD-1*, *CD95*, *LAG-3*, *FOXP3*, and *BACH2* in T cells (73) (Figure 2).

## Single-Gene Defects

Although many human diseases are caused by complex genetic polymorphisms, perhaps the greatest potential for gene therapy is in the ability to treat diseases caused by single Mendelian gene defects. Mutations in genes such as *IFNGR1*, *STAT1*, *IL-12*, and *IL-12R* can lead to immune dysfunction and recurrent mycobacterial infections (74). Genetic disruptions also cause many of the autoinflammatory diseases, such as mutations in the *FMF* gene in FMF, the *LNRP3* gene in NOMID, the *TNFR* gene in TRAPs, and *IL1RN* gene in DIRA. Inserting the corrected sequence for these genes into hematopoietic cells or more safely into differentiated immune cells may result in dramatic improvements in the health of these patients (Figure 2).

## Conclusion

T lymphocytes represent one of the first vehicles to carry therapeutic genes into humans, and its current use, centered on the adoptive transfer of T cells, is proving to be a promising cancer therapeutic modality. However, logistic hurdles still exist for the wider use of this technology due to costs associated with GMP quality viral production and the requirement of significant technologic infrastructure and expertise. Increased collaboration between industry and academia for developing gene therapies may help overcome current financial limitations by developing viable business models.

There exist over 4000 known single-gene disease causing disorders in addition to the innumerable genetic polymorphisms that increase susceptibilities for diseases. Gene therapy in T cells is paving the way for a broader application of this therapeutic modality in human disease. The ability to stably introduce functional genes into hematopoietic stem cells or differentiated cells *ex vivo* provides hope for the thousands of patients diagnosed with a wide range of devastating genetic diseases, highlighted by recent successes in childhood cerebral adrenoleukodystrophy (75) and hemophilia B (76). Gene therapy represents the ultimate form of personalized medicine, and in the future, it is conceivable to imagine that diseases that were once considered untreatable will be readily controlled or eradicated with a single specialized treatment.

## Conflict of Interest Statement

The author declares that the research was conducted in the absence of any commercial or financial relationships that could be construed as a potential conflict of interest.
